# Induction of liver-resident memory T cells and protection at liver-stage malaria by mRNA-containing lipid nanoparticles

**DOI:** 10.3389/fimmu.2023.1116299

**Published:** 2023-08-23

**Authors:** Sayuri Nakamae, Satoshi Miyagawa, Koki Ogawa, Mariko Kamiya, Mayumi Taniguchi, Akari Ono, Maho Kawaguchi, Awet Alem Teklemichael, Jiun-Yu Jian, Tamasa Araki, Yukimi Katagami, Hidefumi Mukai, Takeshi Annoura, Katsuyuki Yui, Kenji Hirayama, Shigeru Kawakami, Shusaku Mizukami

**Affiliations:** ^1^ Department of Immune Regulation, Shionogi Global Infectious Diseases Division, Institute of Tropical Medicine, Nagasaki University, Nagasaki, Nagasaki, Japan; ^2^ Laboratory for Drug Discovery and Disease Research, SHIONOGI & CO., LTD., Osaka, Japan; ^3^ Department of Pharmaceutical Informatics, Graduate School of Biomedical Sciences, Nagasaki University, Nagasaki, Nagasaki, Japan; ^4^ Department of Parasitology, National Institute of Infectious Diseases, Shinjuku-ku, Tokyo, Japan; ^5^ Shionogi Global Infectious Diseases Division, Institute of Tropical Medicine, Nagasaki University, Nagasaki, Nagasaki, Japan; ^6^ School of Tropical Medicine and Global Health, Nagasaki University, Nagasaki, Nagasaki, Japan

**Keywords:** liver-stage malaria, vaccine, mRNA, lipid nanoparticles, resident memory T cells, cellular immunity

## Abstract

Recent studies have suggested that CD8^+^ liver-resident memory T (T_RM_) cells are crucial in the protection against liver-stage malaria. We used liver-directed mRNA-containing lipid nanoparticles (mRNA-LNPs) to induce liver T_RM_ cells in a murine model. Single-dose intravenous injections of ovalbumin mRNA-LNPs effectively induced antigen-specific cytotoxic T lymphocytes in a dose-dependent manner in the liver on day 7. T_RM_ cells (CD8^+^ CD44^hi^ CD62L^lo^ CD69^+^ KLRG1^-^) were induced 5 weeks after immunization. To examine the protective efficacy, mice were intramuscularly immunized with two doses of circumsporozoite protein mRNA-LNPs at 3-week intervals and challenged with sporozoites of *Plasmodium berghei* ANKA. Sterile immunity was observed in some of the mice, and the other mice showed a delay in blood-stage development when compared with the control mice. mRNA-LNPs therefore induce memory CD8^+^ T cells that can protect against sporozoites during liver-stage malaria and may provide a basis for vaccines against the disease.

## 1 Introduction

Malaria is a serious life-threatening infectious disease. In 2020, the World Health Organization (WHO) estimated that there were 241 million cases and 627,000 deaths caused by malaria worldwide (1). Immediately after entering the human body through a mosquito bite, *Plasmodium* sporozoites invade hepatocytes and proliferate. This is known as the liver stage of malaria and is followed by the blood stage. Malaria control strategies include various approaches, such as integrated vector control, clinical management by antimalarial drugs, and preventive vaccines (2–6). However, the rapid development and spread of resistant strains of mosquitoes to insecticides and *Plasmodium* parasites to antimalarial drugs pose a threat to malaria control outcomes (7). The generation of alternative tools, including novel vaccines, is therefore needed. In 2021, the WHO recommended RTS,S/AS01 as the first malaria vaccine for children in sub-Saharan Africa and other regions with a moderate-to-high prevalence of *P. falciparum* infections (1). However, the vaccine has only exhibited modest efficacy and short-term durability and needs to be administered in four doses to achieve a maximum efficacy of 60–70% in terms of reducing clinical complications (8–10). A next-generation vaccine with promising efficacy against infections caused by *Plasmodium* parasites is therefore urgently needed.

In liver-stage malaria, CD8^+^ T cells play an important role in protection, as shown by the radiation-attenuated sporozoite (RAS) vaccine, which has been highly effective in mouse and human malaria models (11–15). The circumsporozoite protein (CSP) has been described as a major antigen in liver-stage malaria in mouse models, and its MHC I-restricted epitope has further been described (16–18). The RTS,S vaccine has been developed for pre-erythrocytic stage interruption using CSP but has been shown to stimulate the production of specific antibodies and weak T cell-mediated immunity (19). Therefore, a malaria vaccine that can effectively induce potent cytotoxic CD8^+^ T cells that are specific to the liver stage is required (20).

Resident memory CD8^+^ T (T_RM_) cells constitute a recently-identified lymphocyte lineage that occupies tissue without recirculating (21). T_RM_ cells are the first-line defenders against reinfection by pathogens. T_RM_ cells further express unique surface markers, such as CD69, based on their localization (21–23). Recently, liver-resident T_RM_ cells have been reported to play an important role in the protection against sporozoite infections (22). The efficient induction of T_RM_ cells in the liver may be key for the development of next-generation malaria vaccines since T_RM_ cells contribute to the protection against sporozoite infections.

Nucleic acid-based therapies have become a new trend in alternative vaccine development (24). Messenger RNA (mRNA)-based vaccinations do not pose the risks of vaccine-derived infections and insertional mutagenesis as opposed to virus-based and DNA vaccines, respectively. mRNA delivery systems to the cytoplasm of target cells have been intensively studied to protect mRNA from RNases. Among them, lipid nanoparticles (LNPs) are the most advanced mRNA carriers intended for vaccine-based therapies (25–27). The ionizable lipids in LNPs form complexes with mRNA in lipid vesicles to yield core-shell structures (28). After LNPs are taken into cells via endocytosis, the ionizable lipids in the LNPs become positively charged in response to acidification. Membrane destabilization is then promoted, and endosomal escape is facilitated to deliver the mRNA in the LNPs to the cytosol. It has also been suggested that LNPs accumulate in the liver when administered systemically via intravenous and intramuscular injections (29, 30). Through such mechanisms, LNPs can deliver intact mRNA to the cytosol for translation in the liver.

To develop a liver-stage-specific T cell-mediated vaccine, we used a liver-directed LNP-based mRNA vaccine platform and the rodent malaria parasite *P. berghei* ANKA (PbA). The mRNA-LNP vaccine was found to induce potent T_RM_ cells in the liver, which are protective against liver-stage malarial infections.

## 2 Materials and methods

### 2.1 Animals

C57BL/6N (B6) and BALB/c mice (5–9 weeks old; CLEA, Tokyo, Japan) were used for the experiments. H-2K^b^-restricted OT-I transgenic mice expressing the TCR specific for SIINFEKL were provided by Dr. H. Kosaka (Osaka University, Osaka, Japan) (31). B6.SJL-Ptprc congenic (B6.SJL) mice (CD45.1^+^) were provided by Dr. Y. Takahama (University of Tokushima, Tokushima, Japan). The B6.SJL and OT-I mice were bred, and the offspring were intercrossed to obtain CD45.1^+^ OT-I mice. The mice were housed in a standard clean room under conventional conditions at the Laboratory Animal Center for Animal Research at Nagasaki University. The animal experiments were approved by the Institutional Animal Care and Use Committee of Nagasaki University and were conducted in accordance with the guidelines for Animal Experimentation at Nagasaki University.

### 2.2 mRNA

OVA and firefly luciferase (FLuc) mRNA were purchased from TriLink (San Diego, CA, USA). The DNA template vector for the transcription of mRNA, pSHI, was constructed by inserting the sequences of the T7 promoter, 5’ UTR, IgGκ-chain signal sequence, haemagglutinin tag (YPYDVPDYA), EcoRI and BamHI restriction sites, 3’ UTR, 120 adenosines, and SapI restriction site into the pUC-GW-Amp vector (GENEWIZ, South Plainfield, NJ, USA). The truncated CSP sequence (PBANKA_0403200) lacking 1–23 amino acids (aa) in the signal sequence, 93–201 aa in the repeat region, and 319–340 aa in the GPI anchor region was synthesized by Eurofins Genomics (Ebersberg, Germany) and inserted into pSHI via the traditional cloning method using the EcoRI and BamHI restriction enzymes (New England Biolabs, Ipswich, MA, USA). The codon usage of the sequences coding the IgGκ-chain signal sequence, haemagglutinin tag, and CSP was optimized for *Mus musculus*. The constructed CSP template plasmid was purified from *Escherichia coli* using the EndoFree Plasmid Maxi Kit (Qiagen, Hilden, Germany) and linearized using the SapI restriction enzyme (New England Biolabs). *In-vitro* transcription was performed using the HiScribe T7 High Yield RNA Synthesis Kit (New England Biolabs) with CleanCap Reagent AG (TriLink) to cap the 5’ end of the RNA. CSP mRNA was purified using LiCl precipitation after the DNase treatment. Ψ-CSP mRNA was synthesized with N^1^-methylpseudouridine-5’-triphosphate (TriLink) and purified using RNeasy Mini Kit (Qiagen).

### 2.3 Preparation of LNP-encapsulated mRNA

LNP-encapsulated mRNA was prepared by mixing lipids and mRNA using a microfluidic system, as previously described (32). COATSOME^®^ SS-OP (NOF, Tokyo, Japan), 1,2-dioleoyl-sn-glycero-3-phosphocholine (DOPC; NOF), cholesterol (Nacalai Tesque, Kyoto, Japan), and 1,2-dimyristoyl-rac-glycero-3-methylpolyoxyethylene (DMG-PEG2000; NOF) were dissolved to 4.5 mM in ethanol. The molar ratio of each lipid was 6/1/3 (SS-OP/DOPC/cholesterol), and DMG-PEG2000 was added at a concentration of 1.5% of the total lipids. The mRNA was diluted to 7.5 μg/mL in 20 mM malic acid buffer (pH 3.0). Lipid and mRNA solutions were mixed in a NanoAssemblr^®^ Benchtop (Precision NanoSystems, British Columbia, Canada) using a total flow rate of 4 mL/min and a flow rate ratio of 3:1 (mRNA:lipid). The resultant solution was dialyzed against MES buffer (pH 6.5) to remove ethanol and then concentrated to obtain the necessary concentration through ultrafiltration using Amicon^®^ Ultra-15 (Merck, Darmstadt, Germany). The LNPs were then suspended in phosphate-buffered saline (PBS).

### 2.4 Characterization of LNPs

The particle size, polydispersity index, and zeta potential of the LNPs were measured using a Zetasizer Nano ZS (Malvern Instruments, Malvern, UK) as previously described (32). The LNPs were diluted 40 times in PBS and introduced into capillary cells. The measurements were then taken at 25°C. To evaluate encapsulation efficiency, mRNA was quantified using the Quant-iT RiboGreen RNA Assay Kit (Thermo Fisher Scientific, Waltham, MA, USA). The unencapsulated mRNA concentration was measured by quantifying the intact LNPs, while the total mRNA concentration was measured after the LNPs had been solubilized using Triton X-100. Encapsulation efficiency (EE) was calculated using the following formula:


EE=[(total mRNA)–(unencapsulated mRNA)] / (total mRNA)

### 2.5 *In-vivo* distribution of FLuc mRNA-LNPs

To assess the changes in the FLuc mRNA-LNPs over time, BALB/c mice were inoculated with FLuc mRNA-LNPs (5 μg) intravenously, intramuscularly, or subcutaneously. To detect luminescence, the mice were administered 150 mg/kg of D-luciferin (Syd labs, Hopkinton, MA, USA) intraperitoneally and anaesthetized with a mixture of oxygen and isoflurane (Wako, Osaka, Japan). Ten minutes after the administration of D-luciferin, the mice were imaged using IVIS Lumina II (Caliper Life Sciences, Waltham, MA, USA) with an exposure time of 5 s. To detect the tissue distribution of the FLuc mRNA-LNPs, B6 mice were intravenously inoculated with FLuc mRNA-LNPs (5 μg) and administered D-luciferin (150 mg/kg) intraperitoneally 3 h later. The reaction was observed for 10 min. The brains, hearts, livers, spleens, lungs, kidneys, and intestines were collected immediately and imaged using an IVIS imager for 5 s. Bioluminescent signals in the regions of interest were quantified using Living Image 3.0 (Caliper Life Sciences).

### 2.6 Adaptive transfer and immunization

OT-I CD8^+^ cells were isolated using the BD™ IMag cell separation system as previously described (33). In brief, CD8^+^ (> 95%) cells were prepared from the spleen, brachial, and inguinal lymph nodes of the CD45.1^+^ OT-I mice using anti-CD8 IMag (BD Biosciences, Franklin, NJ, USA) and injected into the tail veins of the B6 mice (1 × 10^6^/mouse). Two days later, the mice were immunized with OVA mRNA-LNPs intravenously, intramuscularly, or subcutaneously. The BALB/c mice were intramuscularly immunized with 3.35 μg of CSP mRNA-LNPs or 6.7 μg of Ψ-CSP mRNA-LNPs at 3-week intervals.

### 2.7 Tissue processing for T-cell analysis

The mice were anaesthetized with a combination of 0.75 mg/kg of medetomidine hydrochloride (Kyoritsu Seiyaku, Tokyo, Japan), 4 mg/kg of midazolam (Sandoz K.K., Tokyo, Japan), and 5 mg/kg of butorphanol tartrate (Meiji Seika Pharma Co., Ltd., Tokyo, Japan), in addition to the inhalation of isoflurane (Wako) at different time points after being immunized. The mice were perfused with 20 mL of cold PBS, after which their livers and spleens were harvested. The livers were sliced using scissors and crushed in a Petri dish with a syringe plunger. Liver cell suspensions were passed through a 196-μm stainless mesh. After centrifugation at 430 × g for 5 min at room temperature, the pellets were suspended in a solution of 35% isotonic Percoll (GE Healthcare, Chicago, IL, USA). The cells were then centrifuged at 500 × g for 30 min at room temperature with no brake. Parenchymal cells and debris were removed using a disposable pipette, and the pellet was suspended in Gey’s solution to lyse the red blood cells. Liver cell suspensions were then passed through a 70-μm mesh. The spleens were crushed in a Petri dish using a syringe plunger. Spleen cell suspensions were then passed through a 70-μm mesh and treated with Gey’s solution to lyse the red blood cells.

### 2.8 Flow cytometry analysis

The cells were stained with Zombie-Aqua (BioLegend, San Diego, CA, USA) for 15 min at room temperature in the dark to identify and exclude dead cells from the analysis. After being washed, the cells were stained with T-Select H-2K^b^ OVA Tetramer-SIINFEKL-PE (MBL, Tokyo, Japan) or T-Select H-2K^d^ malaria Pb9 Tetramer-SYIPSAEKI-PE (MBL) for 30 min at 4°C in the dark. The cells were then washed and stained for 30 min at 4°C in the dark to identify cell surface molecules with monoclonal antibodies. The antibodies included APC-anti-CD3e (145-2C11), BV785-anti-CD3e, FITC-anti-CD8 (KT15), APC-R700-anti-CD44 (IM7), BV421-anti-CD62L (MEL-14), BV785-anti-CD127 (A7R34), APC-anti-CD69 (H1.2F3), and BV650-anti-KLRG1 (2F1). All of the antibodies were purchased from BioLegend, BD Biosciences, or MBL. The stained cells were fixed with 1% paraformaldehyde (Wako) and analyzed using the BD FACSCelesta Flow Cytometer (BD Biosciences) and FlowJo software (BD Biosciences).

Intracellular staining was performed according to the manufacturer’s instructions (BD Biosciences). The cells (1.5–3 × 10^6^/mL) were stimulated for 6 h with 1 mg/mL of the SIINFEKL peptide in RPMI-1640 medium with L-Glutamine and Phenol Red (Wako) supplemented with 10% heat-inactivated fetal bovine serum (FBS), penicillin/streptomycin, non-essential amino acids (0.1 mM), sodium pyruvate (1 mM), and 2-mercaptoethanol (5 × 10^-5^ M) with GolgiPlug (BD Biosciences) for the last 4 h. The cells were then stained with Zombie-Aqua for 15 min at room temperature in the dark to identify and exclude dead cells from the analysis. After adding the Fc receptor blocker (2.4G2), the cells were stained with the BV785-anti-CD3e, FITC-anti-CD8, and BV421-anti-CD107a monoclonal antibodies or BV421-Rat IgG2b Isotype Control. The cells were fixed, permeabilized with the BD Cytofix/Cytoperm Fixation/Permeabilization kit (BD Biosciences), and stained with APC-anti-IFN-γ (XMG1.2), BV650-anti-TNF-α (MP6-XT22), APC, PE-anti-Granzyme B (QA16A02), or their isotype controls. The cells were then analyzed using FACSCelesta and the FlowJo software.

### 2.9 Serum ALT levels and anti-OVA IgG titers

The serum alanine aminotransferase (ALT) levels of the immunized mice were measured using an automatic analyzer (Fuji DRI-chem 3500V; FUJIFILM, Tokyo, Japan). The levels of anti-OVA IgG antibodies in the sera were determined using an enzyme-linked immunosorbent assay (ELISA), as previously described (34). In brief, ELISA plates were coated with 100 μg/well of the OVA protein (Wako) in PBS overnight at 4°C. The coated plates were washed with PBS containing 0.05% Tween 20 (washing buffer) and blocked with PBS containing 10% FBS for 30 min at room temperature. After the wash, serum (×40) was added to the plates, which were then incubated overnight at 4°C. The plates were washed, incubated with biotin-conjugated goat anti-mouse IgG-Fc fragment antibodies (Bethyl Laboratories, Montgomery, TX, USA) for 1 h at room temperature, washed, and then incubated with horseradish peroxidase-conjugated streptavidin (BioLegend) for 30 min at room temperature. After the wash, 1×TMB substrate solution (Thermo Fisher Scientific) was added to each well, and the plates were incubated at room temperature for 15 min. Phosphoric acid (2 M) was used to stop the reaction, and the absorbance was read at 450 and 570 nm using an iMark Microplate Absorbance Reader (Bio-Rad, Hercules, CA, USA).

### 2.10 Parasites and infection

Recombinant *Plasmodium berghei* ANKA (PbA) expressing GFP (PbA-GFP) and recombinant *P. berghei* 676m1cl1 line (Pb-lucGFP) were used (35–37). Sporozoites were obtained from the salivary glands of infected female *Anopheles stephensi* mosquitoes 21–23 days after blood meal feeding. The mice were intravenously infected with 200 PbA-GFP sporozoites, as previously described (38). Parasitaemia levels were determined based on the expression of GFP using a BD FACSCelesta Flow Cytometer 3 days after the infections. Another group of mice were intravenously infected with 3000 Pb-lucGFP sporozoites. Forty-four hours after infection, the mice were administered 150 mg/kg of D-luciferin intraperitoneally and anaesthetized with a mixture of oxygen and isoflurane. Ten minutes later, the mice were imaged using IVIS Lumina II with an exposure time of 3 min. Bioluminescent signals in the regions of interest were quantified using Living Image 3.0. The levels of parasitemia were determined using a microscopic examination of standard thin blood smears stained with Giemsa.

### 2.11 CD8 depletion

Each mouse was intraperitoneally administered 100 μg of anti-mouse CD8a antibody (2.43) or rat IgG2b isotype control (LTF-2) 1 day before the induction of the sporozoite infections. Both antibodies were purchased from Bio X Cell (Lebanon, NH, USA).

### 2.12 Statistical analysis

Statistical analyses were performed using the GraphPad Prism software (version 8). All data were tested for normal distribution using the Shapiro–Wilk test. For comparisons between two groups, Welch’s t-test was used to assess statistical significance if the sample data followed a normal distribution; otherwise, the Mann–Whitney U test was used. For comparisons between more than two groups, a one-way or two-way ANOVA with Bonferroni’s *post-hoc* test was performed if the sample data followed a normal distribution; otherwise, the Kruskal–Wallis and Dunn’s *post-hoc* tests were performed.

## 3 Results

### 3.1 Preferential expression of mRNA carried by LNPs in the liver

**Figure 1:**
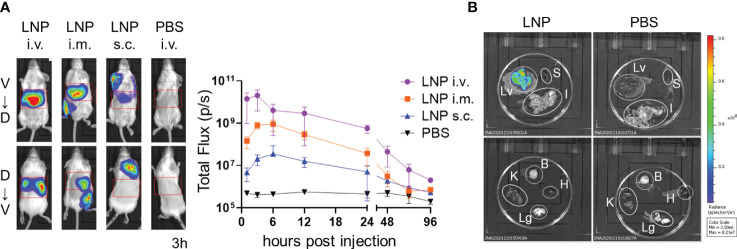
Distribution of LNPs in the mice. **(A)** Temporal expression of FLuc mRNA. BALB/c mice were injected with LNPs containing 5 µg of FLuc mRNA (5 µg of FLuc mRNA-LNPs) intravenously, intramuscularly, or subcutaneously (n = 3). As a negative control, another group of mice were intravenously injected with 100 μL of PBS. Bioluminescence was measured 1, 3, 6, 12, 24, 48, 72, and 96 h post injection. Representative images of ventrodorsal (V→D) and dorsabdominal (D→V) shootings taken 3 h after the injection are shown on the left. A summary graph of the total flux [photons/s] in the liver is shown on the right. Data represent the mean ± SD. **(B)** FLuc expression in organs. B6 mice were intravenously injected with LNPs containing 5 μg of FLuc mRNA or 200 μL of PBS (n = 4). The liver, spleen, gastrointestinal tract, kidneys, brain, heart, and lungs were collected 3 h after injection, and bioluminescence was measured. Representative images for each organ are shown. Lv, Liver; S, Spleen; I, gastrointestinal tract; B, Brain; K, Kidney; H, Heart; Lg, Lung.

We generated LNPs composed of four types of lipids, including SS-cleavable pH-activated lipid-like material (ssPalm), 1,2-dioleoyl-sn-glycero-3-phosphocholine (DOPC), 1,2-dimyristoyl-rac-glycero-3-methylpolyoxyethylene (DMG-PEG2000), and cholesterol. ssPalm contains dual sensing motifs that consist of a tertiary amine and disulfide bond that can respond to the intracellular environment, such as acidic conditions in the endosome and reductive conditions in the cytosol (39). We prepared LNPs with SS-OP, which is the third generation of ssPalm and has an oleic acid scaffold and a phenyl ester linker with anti-inflammatory and self-degradable properties (39). First, we used LNPs carrying firefly luciferase (FLuc) reporter mRNA (FLuc mRNA-LNPs) to determine the target tissue of the LNPs (**Table 1**). To determine the distribution of the LNPs, BALB/c mice were used as the pigment from darker-haired small animals can reduce bioluminescent signals. Three groups of mice were injected with FLuc mRNA-LNPs intravenously, intramuscularly, or subcutaneously, and the bioluminescence intensity of the total body and upper abdominal regions were examined 1–96 h after the injections (**Figure 1A**). The mice that received an intravenous injection exhibited the strongest FLuc expression in the liver, with a peak response 3 h after the injection. The mice that received intramuscular and subcutaneous injections exhibited lower FLuc expression than those receiving intravenous injections, with a delayed peak 6 h after the injections. FLuc expression levels were higher after the intramuscular injections than after the subcutaneous injections. Following the intramuscular and subcutaneous injections, FLuc expression was detected around the administration sites. To assess the immune responses in the mice, we used ovalbumin (OVA) as a model antigen. OVA has a dominant CD8^+^ T cell epitope that is restricted by H-2k^b^. We removed each organ from the C57BL/6N (B6) mice that had been administered FLuc mRNA-LNPs intravenously and performed an *ex-vivo* imaging analysis 3 h after the injections (**Figure 1B** and **Supplementary Figure 1**). The FLuc signals in the liver showed the highest intensity, whereas those in the spleen, intestines, and kidney were marginal.

**Table 1:** Physicochemical properties of LNPs.

Size (z-average) (nm)	PDI^*^	Zeta potential (mV)	EE^**^ (%)
94.8 ± 2.7	0.070 ± 0.012	–2.13 ± 0.84	97.1 ± 1.61

^*^PDI, polydispersity index; ^**^EE, Encapsulation efficiency. Data represent mean ± S.D. (n = 3).

### 3.2 Intravenous injections of OVA mRNA-LNPs activated OVA-specific CD8^+^ T cells and induced resident memory CD8^+^ T cells in the liver

**Figure 2:**
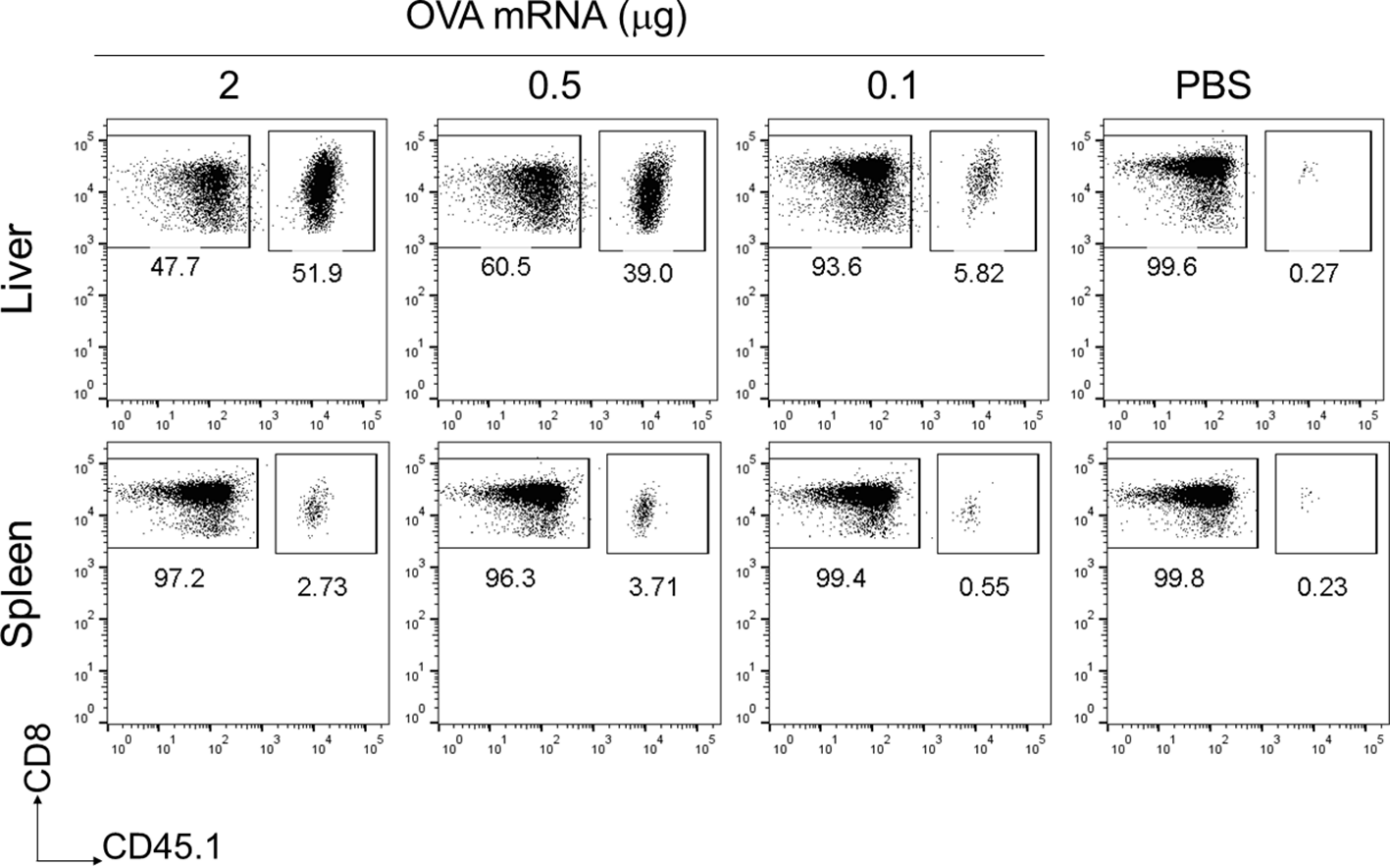
OT-I cells were strongly activated in the liver following a single intravenous injection of OVA mRNA-LNPs. CD45.1^+^ OT-I cells (1 × 10^6^) were adoptively transferred to B6 mice. Two days after cell transfer, the mice were intravenously injected with serial doses of OVA mRNA-LNPs or PBS (n = 3). Seven days post injection, the mice were analyzed. Representative dot plots show the proportion of OT-I in CD3^+^CD8^+^ cells within the liver (upper panel) and spleen (lower panel).

**Figure 3:**
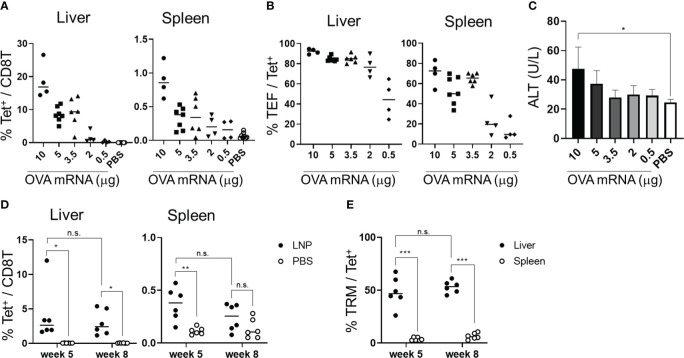
OVA mRNA-LNPs induced OVA-specific CD8^+^ T cells and memory cells in the liver. **(A–C)** B6 mice were intravenously injected with the indicated doses of OVA mRNA-LNPs or 200 μL of PBS. Seven days after injection, the mice were analyzed. The proportion of H-2K^b^ SIINFEKL-restricted tetramer^+^ within the CD8^+^ T cells in the liver (left) and spleen (right) is shown **(A)**. The proportion of effector (CD127^-^ CD44^hi^ CD62L^lo^) cells within the tetramer^+^ cells in the liver (left) and spleen (right) is shown **(B)**. The serum ALT levels on day 7 are shown in **(C)**. **(D, E)** B6 mice were intravenously injected with 5 μg of OVA mRNA-LNPs or 200 μL of PBS (n = 6). One and two months after injection, the mice were analyzed. The proportion of tetramer^+^ CD8^+^ T cells in the liver (left) and spleen (right) is shown **(D)**. The proportion of T_RM_ (CD44^hi^ CD62L^lo^ CD69^+^ KLRG1^-^) cells within the tetramer^+^ cells in the liver (left) and spleen (right) is shown **(E)**. These results were obtained from three **(A–C)** and two experiments **(D, E)**. The data represent the mean **(A, B, D, E)** or the mean ± SD **(C)**; *P< 0.05, ***P< 0.001, n.s. (not significant), as indicated by a one-way ANOVA with Bonferroni’s post-hoc test **(C)** and Mann–Whitney U test **(D, E)**.

To assess the immunogenicity of the mRNA-LNPs in the liver, we used OVA-specific T-cell receptor (TCR) transgenic mice, hereafter referred to as “OT-I” mice (31). B6 mice were administered 1 × 10^6^ CD8^+^ T cells from CD45.1^+^ OT-I mice (OT-I cells) and intravenously immunized with OVA mRNA-LNPs (n = 3). Seven days after immunization, the proportion of OT-I cells within the CD8^+^ T cells reached 56.6 ± 8.3% in the livers of the mice immunized with 2 μg of OVA mRNA (**Figure 2**). Although the OT-I cells increased in both the liver and spleen in a dose-dependent manner, their increase was greater in the liver than in the spleen (**Figure 2**). These results indicate that OVA mRNA-LNPs activate OT-I cells and induce their proliferation. After confirming the immunization effects of the mRNA-LNPs in the liver, we examined whether the OVA mRNA-LNPs would induce endogenous OVA-specific CD8^+^ T cells in B6 mice. Seven days after immunization, tetramer-positive OVA-specific CD8^+^ T cells and their CD44^hi^ CD62L^lo^ CD127^-^ effector phenotypes increased in both the liver and spleen in a dose-dependent manner. The numbers of tetramer-positive cells and their effector phonotypes in the liver were greater than those in the spleen (**Figures 3A, B** and **Supplementary Figures 2A, 3A, B**). Serum alanine aminotransferase (ALT) levels were further measured to evaluate the extent of liver injury in the immunized mice. Although the ALT levels in the mice immunized with 10 μg of OVA mRNA were higher than those in the control mice, no significant differences were observed between the mice immunized with 5 μg of OVA mRNA or less and the control mice (**Figure 3C**). We therefore used 5 µg of OVA mRNA-LNPs for the subsequent experiments.

To assess whether the OVA mRNA-LNPs could induce OVA-specific T_RM_ cells, B6 mice were immunized once with OVA mRNA-LNPs intravenously. One month after immunization, OVA tetramer^+^ CD8^+^ T cells remained in the liver and spleen, with their proportion in the liver being higher than that in the spleen (**Figure 3D**). T_RM_ cells (CD8^+^ CD44^hi^ CD62L^lo^ CD69^+^ KLRG1^-^) comprised 48.4 ± 13.1% of the OVA tetramer^+^ CD8^+^ T cells in the liver, but there were few T_RM_ cells in the spleen (**Figure 3E** and **Supplementary Figures 2B, 3C, D**). Effector memory CD8^+^ T cells (T_EM_) (CD8^+^ CD44^hi^ CD62L^lo^ CD69^-^) comprised 37.9 ± 14.9% and central memory CD8^+^ T cells (T_CM_) (CD8^+^ CD44^hi^ CD62L^hi^) comprised 6.8 ± 3.5% of the tetramer^+^ CD8^+^ T cells in the liver (**Supplementary Figures 3C, D**). In the spleen, OVA-specific CD8^+^ T cells consisted of 3.7 ± 1.4% T_RM_ cells, 50 ± 7.3% T_EM_ cells, and 33.3 ± 10.5% T_CM_ cells. The number of liver T_RM_ cells was maintained for more than 2 months after immunization (**Figures 3D, E**). To investigate the effector function of the memory CD8^+^ T cells in the liver, we performed intracellular cytokine staining 1 month after immunization (**Supplementary Figure 4**). An *in-vitro* stimulation with the SIINFEKL peptide, which is an MHC class I dominant epitope of OVA, detected IFN-γ^+^ and INF-γ^+^ TNF-α^+^ double-producing CD8^+^ T cells in the livers of the immunized mice (**Supplementary Figure 4A**). In addition, the co-expression of Granzyme B and the degranulation marker CD107a was induced in the CD8^+^ T cells in response to the SIINFEKL peptide stimulation (**Supplementary Figure 4B**). Collectively, these data suggest that a single dose of OVA mRNA-LNPs can induce OVA-specific T_RM_ cells, which may possess multifunctional effector activity.

**Figure 4:**
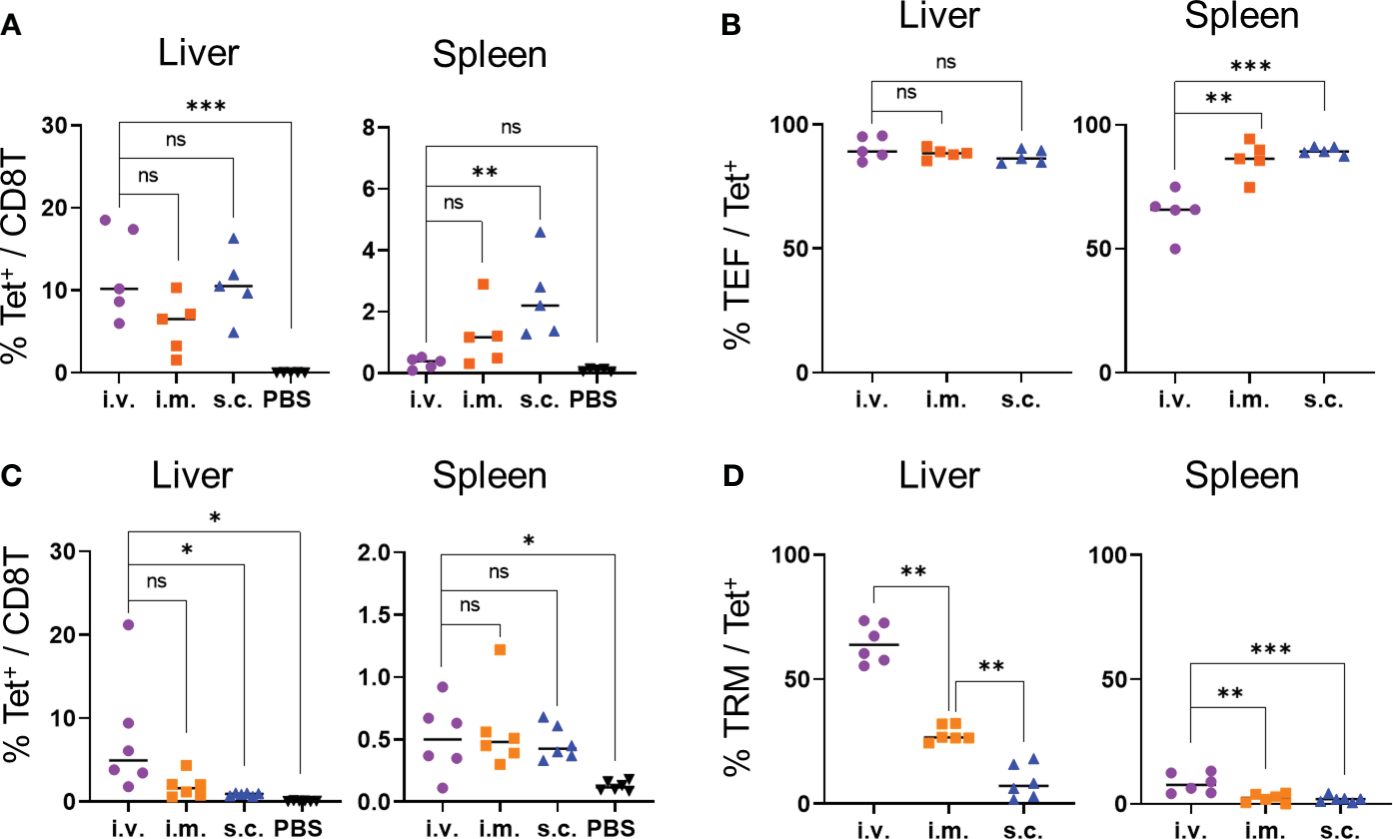
T_RM_ cell generation was affected based on the injection routes of the OVA mRNA-LNPs. B6 mice were injected with 5 μg of OVA mRNA-LNPs intravenously (i.v.), intramuscularly (i.m.), or subcutaneously (s.c.). As a negative control, another group of mice were intravenously injected with 100 μL of PBS. **(A, B)** Seven days after injection, the mice were analyzed (n = 5). The proportion of tetramer^+^ CD8^+^ T cells in the liver (left) and spleen (right) is shown in **(A)**. The effector (CD127^-^ CD44^hi^ CD62L^lo^) cells within the tetramer^+^ cells in the liver (left) and spleen (right) are shown **(B)**. **(C, D)** One month after injection, the mice were analyzed (n = 6). The proportion of tetramer^+^ CD8^+^ T cells in the liver (left) and spleen (right) is shown **(C)**. T_RM_ (CD44^hi^ CD62L^lo^ CD69^+^ KLRG1^-^) cells within the tetramer^+^ cells in the liver (left) and spleen (right) are shown **(D)**. These results were obtained in two experiments. Data represent the mean; *P< 0.05, **P< 0.01, ***P< 0.001, n.s. (not significant), as indicated by a one-way ANOVA with Bonferroni’s *posthoc* test.

Next, we examined whether immunization via intramuscular and subcutaneous routes would induce OVA-specific T_RM_ cells in the liver. Seven days after immunization, the levels of OVA-specific CD8^+^ T cells in the livers of the mice immunized via intramuscular and subcutaneous routes were not significantly different from those immunized intravenously, whereas the levels of OVA-specific CD8^+^ T cells in the spleen were higher in the mice immunized subcutaneously than in those immunized intravenously (**Figure 4A**). The proportions of OVA-specific CD8^+^ T cells with the effector phenotype (CD44^hi^ CD62L^lo^ CD127^-^) that were induced in the liver following intravenous, intramuscular, and subcutaneous immunization were at similar levels (90.4 ± 4.2, 88.38 ± 1.8, and 87.1 ± 2.5%, respectively; **Figure 4B**, **Supplementary Figures 2A, 5**). After 1 month, the levels of OVA-specific T_RM_ cells were evaluated in the mice immunized via different routes. The proportions of OVA-specific CD8^+^ T cells in the livers of the intravenously-immunized mice were comparable to those in the intramuscularly-immunized mice and higher than those in the subcutaneously-immunized mice (**Figure 4C**). The proportion of T_RM_ cells in the liver was highest in the mice immunized intravenously, followed by those immunized intramuscularly (**Figure 4D** and **Supplementary Figure 6**). In contrast, the majority of the tetramer^+^ cells in the liver were T_EM_ cells when the mice were immunized subcutaneously (**Supplementary Figure 6B**). In the spleen, OVA-specific memory CD8^+^ T cells were poorly induced by all three forms of immunization and were mainly T_EM_ and T_CM_ cells (**Figures 4C, D**; **Supplementary Figure 6**). Additionally, we tested the levels of IgG specific to the OVA protein to confirm the induction of humoral immunity by the mRNA-LNPs. Compared with the phosphate-buffered saline (PBS) control, the subcutaneous injections induced significantly higher levels of anti-OVA IgG antibodies, whereas the intravenous and intramuscular injections did not (**Supplementary Figure 7**). Taken together, these results indicate that the intravenous route was the most effective in inducing specific T_RM_ cells in the liver, followed by the intramuscular and subcutaneous routes; however, when considering the practical applications of human vaccines, the intramuscular route may be more feasible.

### 3.3 Intramuscular immunization in BALB/c mice with CSP mRNA-LNPs protected against *Plasmodium berghei* ANKA sporozoites

**Figure 5:**
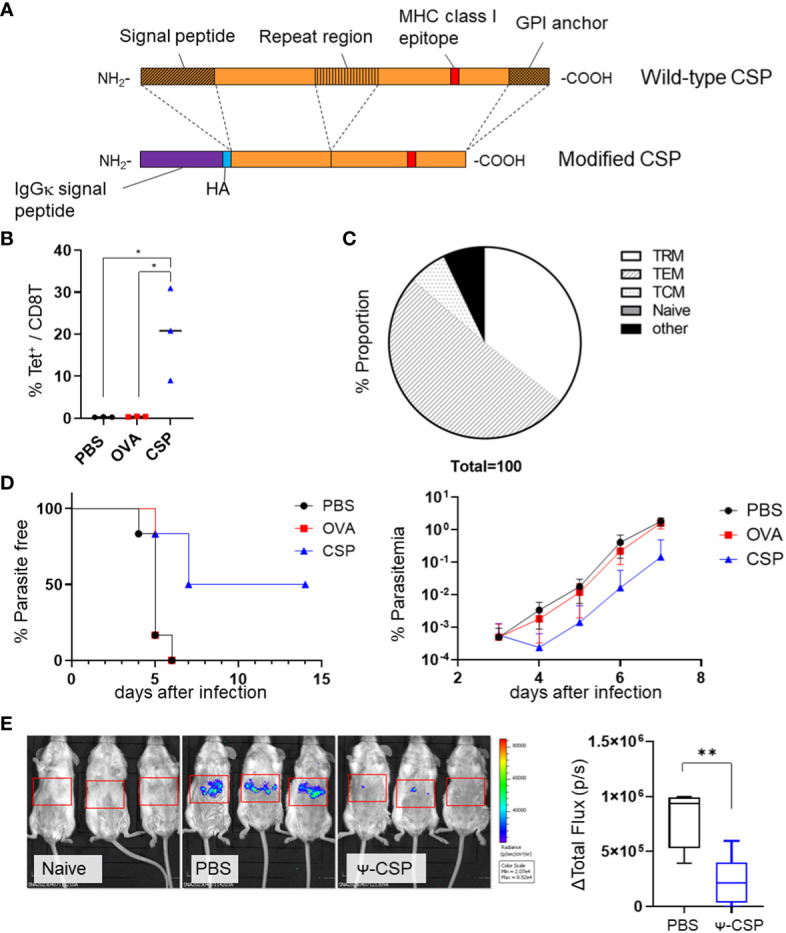
Sterile immunity against the PbA sporozoite was induced by PbA circumsporozoite protein (CSP) mRNA-LNPs. **(A)** Structure of the modified CSP. The signal peptide, repeat region (B-cell epitope), and GPI anchor were deleted from the wild-type CSP, and signal peptides of IgGκ and HA tags were inserted to create modified CSP. **(B)** BALB/c mice were injected with 3.35 μg of CSP, 5 μg of OVA mRNA-LNPs, or 100 μL of PBS intramuscularly twice at 3-week intervals. One month after the final injection, the proportion of tetramer^+^ cells within the CD8^+^ T cells in the liver was observed. **(C)** The proportions of T_RM_ (CD44^hi^ CD62L^lo^ CD69^+^ KLRG1^-^), T_EM_ (CD44^hi^ CD62L^lo^ CD69 ^-^), T_CM_ (CD44^hi^ CD62L^hi^), naïve (CD44^lo^ CD62L^hi^), and other cell populations within the tetramer^+^ cells in the livers of the mice immunized with CSP mRNA-LNPs are shown. **(D)** Mice immunized with CSP mRNA-LNPs were infected with 200 PbA-GFP sporozoites. The left graph shows the proportion of uninfected mice (less than 0.005% of parasitaemia). Parasitaemia 3–7 days after infection is shown on the right side. **(E)** BALB/c mice were intramuscularly injected with 6.7 μg of N1-methyl-pseudouridine CSP (Ψ-CSP) mRNA-LNPs or 100 μL of PBS twice at 3-week intervals (n = 6). One month after final injection, the mice were infected with 3000 Pb-lucGFP sporozoites. Forty-four hours after infection, bioluminescence was measured. Representative images of ventrodorsal shootings are shown on the left. A summary graph of the total flux [photons/s] in the liver is shown on the right. The results were obtained across two experiments. **(B)** The data represent the mean; *P< 0.05, **P< 0.01, Kruskal–Wallis test with Dunn’s *post-hoc* test. **(D)** The data represent the mean of parasitaemia ± SD. The Kruskal–Wallis test with Dunn’s *post-hoc* test was performed 3–7 days after infection. Compared to the PBS group, the PbCSP group exhibited significantly lower parasitaemia 4–7 days after infection (*P<* 0.01). Compared with the OVA group, the PbCSP group exhibited significantly lower parasitaemia 6 and 7 days after infection (*P<* 0.05). **(E)** The data represent the mean ± SD; **P< 0.01, as indicated by a Mann–Whitney U test.

To determine the efficacy of vaccinations with *Plasmodium* antigens and CSPs, we immunized BALB/c mice with PbA CSP mRNA as the dominant MHC class I epitope of PbA CSP is present on H-2K^d^ and not on K^b^ or D^b^ (**Figure 5A**). The NANP repeat region of the CSP was excluded from the construct as we mainly aimed to induce a cell-mediated protective immune response. The BALB/c mice were immunized intramuscularly with CSP mRNA-LNPs twice at 3-week intervals. As a negative control, another group of mice were immunized with OVA mRNA-LNPs. The proportion of CSP-specific CD8^+^ T cells significantly increased in the livers of the CSP mRNA-LNP-immunized mice 33 days after the last immunization; 35.5 ± 7.2% of the cells were T_RM_ cells (**Figures 5B, C** and **Supplementary Figure 8**). One month after the second immunization, the mice were administered with 200 GFP-expressing PbA (PbA-GFP) sporozoites. Half of the immunized mice (three out of six) exhibited sterile immunity, and the onset of the blood stage was delayed by 2 days in the other mice when compared with the negative control mice (**Figure 5D**). To confirm that immunization with CSP mRNA-LNPs provides protection during the liver-stage malaria, the parasite burden in the liver was determined using luciferase- and GFP-expressing *P. berghei* (Pb-lucGFP). BALB/c mice were immunized twice with N1-methyl-pseudouridine CSP (Ψ-CSP) mRNA-LNPs at 3-week intervals and challenged with 3000 Pb-lucGFP sporozoites 5 weeks after the final immunization. Forty-four hours after infection, luciferase activity was monitored using IVIS. The expression levels of luciferase in Ψ-CSP-immunized mice were significantly lower than those in the control mice (**Figure 5E**). We also evaluated the longevity of the efficacy of CSP mRNA-LNPs 9 and 13 weeks after the final immunization (**Supplementary Figure 9**). Protective immunity against the sporozoite infection in the mice immunized with Ψ-CSP persisted for at least 13 weeks after final the immunization.

**Figure 6:**
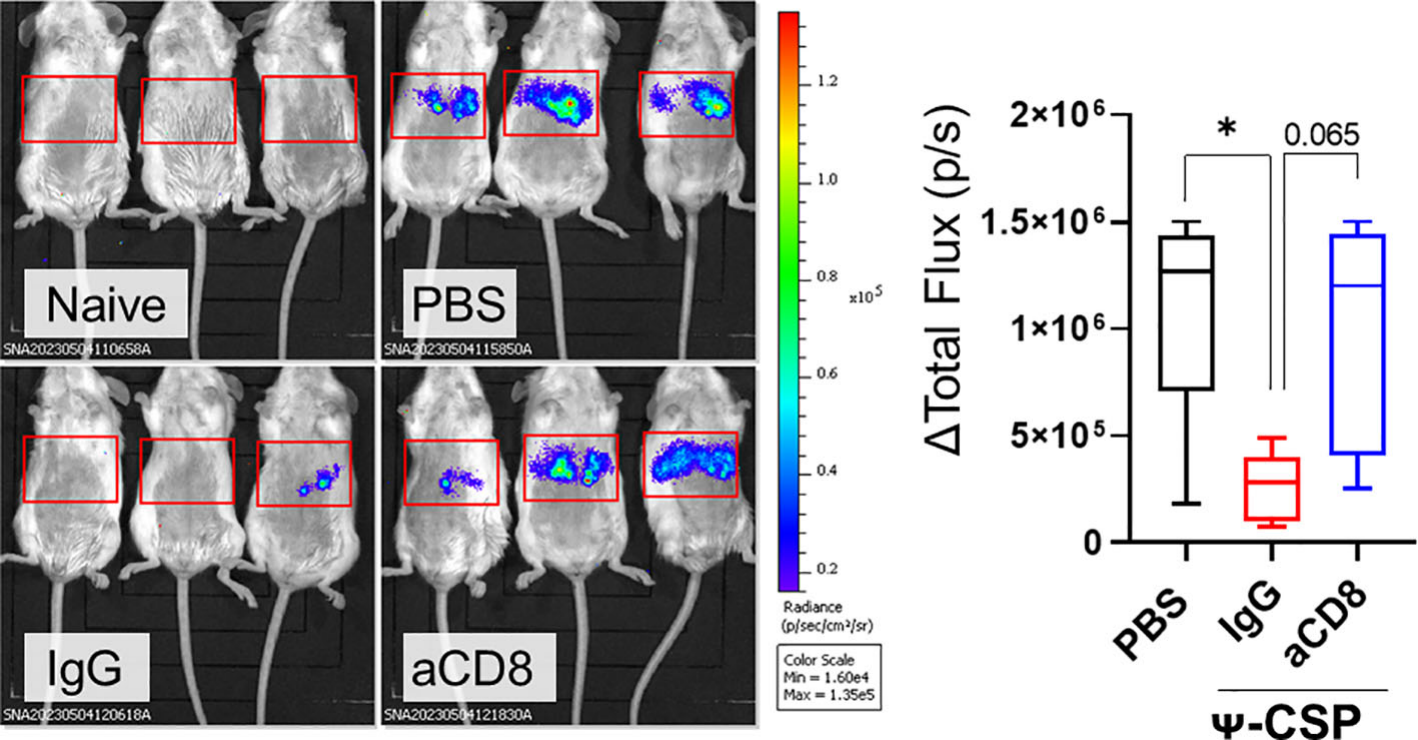
CD8^+^ T cells played a pivotal role of the protection against sporozoite infection in the mice immunize with CSP mRNA-LNPs. BALB/c mice were intramuscularly injected with 6.7 μg of Ψ-PbCSP mRNA-LNPs or 100 μL of PBS twice at 3-week intervals. One month after the final injection, the mice immunized with Ψ-CSP mRNA-LNPs were intraperitoneally administrated 100 μg of anti-CD8 antibodies to deplete CD8^+^ T cells (n = 5). The other mice immunized with Ψ-CSP mRNA-LNPs were intraperitoneally administrated 100 μg of control rat IgG2b as a control (n = 5). One days after the antibody treatment, the mice were infected with 3000 Pb-lucGFP sporozoites. Forty-four hours after infection, bioluminescence was measured. Representative images of ventrodorsal shootings are shown on the left. A summary graph of the total flux [photons/s] in the liver is shown on the right. The results were obtained across three experiments. The data represent the mean ± SD; *P< 0.05, as indicated by a one-way ANOVA with Bonferroni’s *post-hoc* test.

To confirmed that this protection is dependent on CD8^+^ T cells, Ψ-CSP mRNA-LNP immunized mice were administrated anti-mouse CD8 antibodies 1 day before the sporozoite infection to deplete CD8^+^ T cells. As a negative control, Ψ-CSP mRNA-LNP immunized mice were administrated control IgG 1 day before the sporozoite infection. These mice were infected with 3000 Pb-lucGFP sporozoites. Forty-four hours after infection, the control IgG-treated mice exhibited significantly lower levels of luciferase expression than the unimmunized mice (**Figure 6**). In contrast, the anti-CD8-treated mice exhibited luciferase expression levels that were similar to those in the unimmunized mice and higher than those in the control IgG-treated mice, although the latter results were not significant. These results indicate that CD8^+^ T cells played a crucial role in the protection against sporozoite infection in mice immunized with CSP mRNA-LNPs. Taken together, these results suggest that mRNA-LNPs have the potential to be used in malaria vaccines for clinical use.

## 4 Discussion

RAS is a well-established malaria vaccine model that provides sterile protection in rodent models and humans infected with *P. falciparum* (11, 15). The major mechanisms of action of RAS include the induction of cytotoxic T cells residing in the liver, which terminate the liver-stage propagation of *P. falciparum* and antibodies that block the invasion of sporozoites into hepatocytes (19). In this study, we aimed to develop a malaria vaccine that efficiently induces cellular immunity in the liver. We found that mRNA-LNPs targeted the expression of its coding protein in the liver and induced antigen-specific liver T_RM_ cells through intravenous or intramuscular injections. Generally, LNPs are thought to accumulate in the liver when administered systemically via intravenous and intramuscular injections (27, 29, 30). This is likely because the liver is a site for lipid metabolism. It has been shown that liver T_RM_ cells can be induced by targeting antigens to the liver using the “prime and trap vaccination” method, in which antigen-specific CD8^+^ T cells are activated in primary lymphoid organs at the “prime” step and targeted to the liver using antigen peptide-expressing recombinant adeno-associated viruses in the “trap” step (22, 40, 41). mRNA-LNPs target antigens in the liver and are therefore an ideal method of choice to induce T_RM_ cells in the liver.

It has been reported that *P. falciparum* CSP (PfCSP) mRNA-LNP vaccinations substantially protect against infections caused by transgenic *P. berghei* sporozoites expressing PfCSP (42). The mRNA construct in these vaccines includes a NANP B-cell epitope that is intended to provoke an effective antibody, particularly an anti-NANP antibody. In the study that developed the vaccines, the researchers analyzed cytokine production from splenocytes stimulated with PfCSP ex-vivo. In our study, we confirmed that protective immunity can be induced by PbA CSP mRNA without the NANP sequence that includes B-cell epitope and that the protection against sporozoite infection depends on CD8^+^ T cell by treatment with anti-CD8 antibody. Moreover, we observed marked CSP-specific T_RM_ generation in the liver. We focused on the cellular immunity in the liver, especially liver T_RM_.

In our LNPs, SS-OP was utilized as an ionizable lipid that plays an important role in delivering LNPs into the cytoplasm of a target cell. SS-OP is the third generation of ssPalm, which has two types of units composed of a tertiary amine and disulfide bond to accelerate the degradation of LNPs, thereby resulting in the efficient delivery of nucleic acids into the cytosol (39). SS-OP has an oleic acid scaffold and a phenyl ester linker and has exhibited anti-inflammatory and self-degradable properties that allow for the efficient release of mRNA (43, 44). Compared with DLin-MC3-DMA, which is an ionizable lipid that was used in the first-approved siRNA-based drug (ONPATTRO^®^) and has a tertiary amine, SS-OP efficiently delivered mRNA into the liver (43). Our LNPs that were composed of SS-OP likely induced a large amount of antigen expression in the liver without excessive inflammatory responses, which would have caused autoimmunity. Nevertheless, further assessment of our LNPs should be pursued.

The mRNA-LNPs induced stronger immune responses in the liver than in the spleen. This was likely related to the expression levels of the antigens, as shown by their predominant expression in the liver after the intravenous injections of the mRNA-LNPs. This relationship also reflected the route of immunization. One month after immunization, when antigens in the liver were highly expressed, the antigen-specific T_RM_ cells remained in the liver in higher numbers when the mice were immunized via the three different routes. Furthermore, because the intramuscular and subcutaneous injections led to the expression of antigens at the injection sites, these routes likely primed the immune response through other mechanisms, such as different kinds of antigen-presenting cells. The memory phenotype of antigen-specific CD8^+^ T cells and levels of specific antibodies also varied depending on the route of immunization. Further studies on the actual mechanisms that induced these differences are required to better understand these findings.

RAS, genetically-attenuated sporozoites (GAS), and chemically-attenuated sporozoites (CAS) in mice and humans have been reported to provide almost 100% sterile immunity at the pre-erythrocytic stage (15, 45–49). The present forms of mRNA-LNP-coding CSPs do not yield complete sterile immunity. Out of the six mice that were vaccinated with CSP mRNA, three exhibited sterile immunity, and the other three showed development of a blood stage that was delayed by 2 days. This delayed onset of the blood stage was considered to lead to approximately 80% protection against the liver stage of malaria (50). When considering why our LNPs did not yield complete sterile protection, we determined that the number of CSP-specific T_RM_ cells was insufficient, although the possibility of a low function in the T_RM_ cells induced by the mRNA-LNPs has also been taken into account. Previous studies have shown that RASV-immunized B6 mice have approximately 10^4^ of specific T_RM_ cells in the liver and further exhibit 30% sterile immunity (22, 40, 51). This number of T_RM_ cells in the liver was similar to that of our CSP-specific T_RM_ cells in the liver. These studies have further shown that complete protection against PbA sporozoites requires 10^5^ specific T_RM_ cells in the liver. In BALB/c mice, *P. yoelii* RASV, which is a vaccine against another strain of rodent malaria, has been found to induce 70% sterile protection, although only 10^2^ of CSP-specific T_RM_ cells in the liver have been found to be induced following the use of this vaccine (52). However, the required number of T_RM_ cells in the liver is likely to differ depending on the malarial strains causing infection. There may also be other protective factors, such as natural immunity by adjuvant effects. In such cases, even if the same levels of adaptive immunity occur, variation may further occur individually as found in a human study investigating gamma delta T cells, natural killer cells, and CD4^+^ T cells in Controlled Human Malaria Infection (53).

In conclusion, we successfully used the mRNA-LNP platform to induce antigen-specific T_RM_ cells in the liver and further induced strong protective immunity against sporozoite infections. This platform may therefore be useful in the development of new vaccine candidates to induce liver T_RM_ cells efficiently. It may further contribute to accelerating the development of T cell-based malaria vaccines.

## Data availability statement

The raw data supporting the conclusions of this article will be made available by the authors, without undue reservation.

## Ethics statement

The animal study was reviewed and approved by Animal Care and Use Committee of Nagasaki University.

## Author contributions

SN and ShM designed the study. SN, SaM, KO, MKam, MT, AO, MKaw, AT, J-YJ, TAr, and YK conducted the experiments. SN wrote the original manuscript draft. SN, SaM, KO, AT, J-YJ, KY, KH, and ShM wrote the manuscript. All authors have reviewed and approved the final version of the manuscript. This study was supervised by HM, TAn, KY, KH, SK, and ShM. All authors contributed to the article and approved the submitted version.
