# Supplementary material for: Induction of liver-resident memory T cells and protection at liver-stage malaria by mRNA-containing lipid nanoparticles
Source: Front Immunol. 2023 Aug 23;14:1116299. doi: 10.3389/fimmu.2023.1116299 (PMC10482405; doi:10.3389/fimmu.2023.1116299)
Supplement: Supplementary file 1 [file DataSheet_1.pdf]

Supplementary Material

Induction of liver-resident memory T cells and protection at liver-stage malaria by mRNA-containing lipid nanoparticles

Sayuri Nakamae, Satoshi Miyagawa, Koki Ogawa, Mariko Kamiya, Mayumi Taniguchi, Akari Ono, Maho Kawaguchi, Awet Alem Teklemichael, Jiun-Yu Jian, Tamasa Araki, Yukimi Katagami, Hidefumi Mukai, Takeshi Annoura, Katsuyuki Yui, Kenji Hirayama, Shigeru Kawakami, Shusaku Mizukami*

*** Correspondence:** mizukami@nagasaki-u.ac.jp

Supplementary Figures

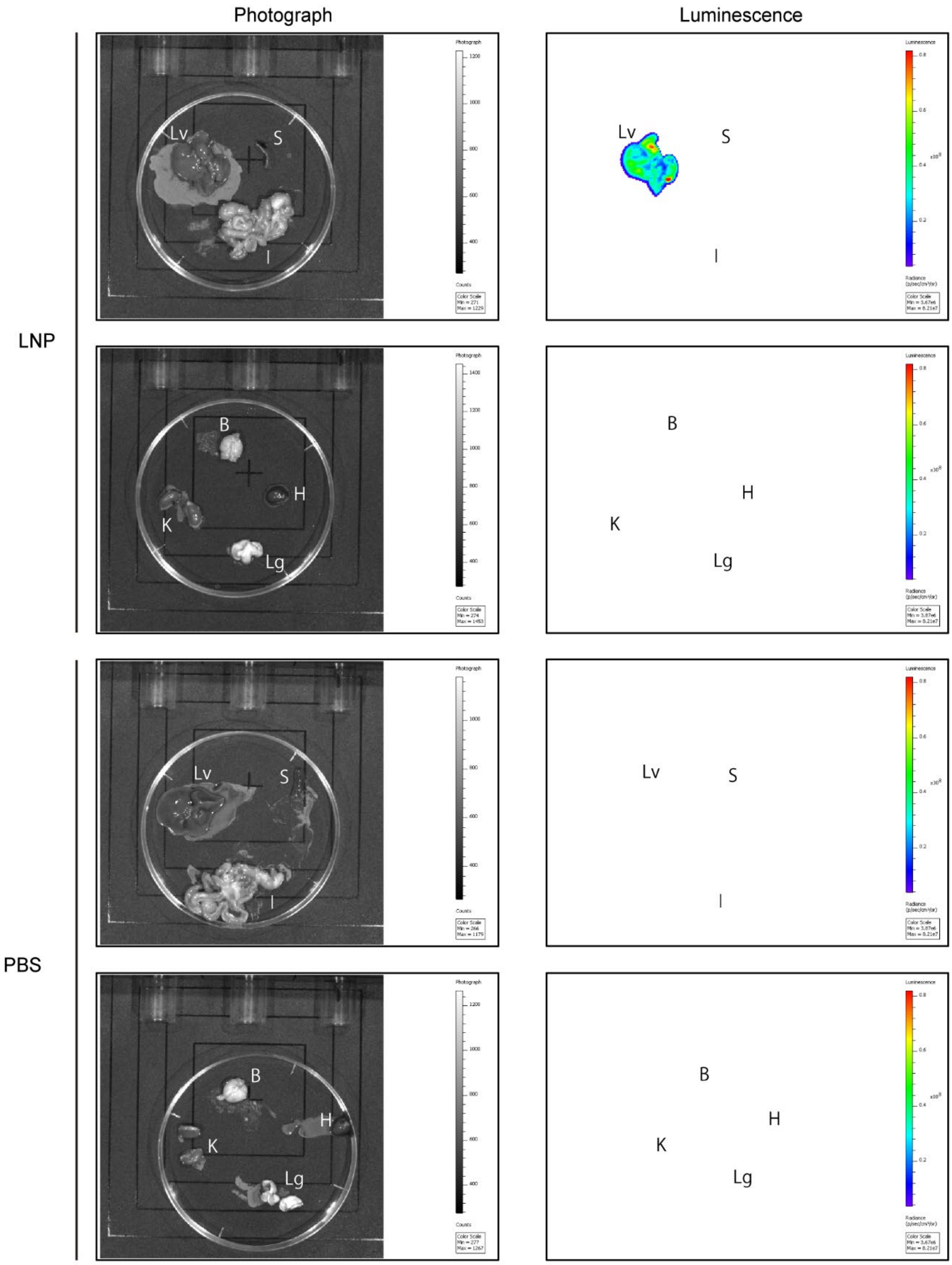

Supplementary Fig. 1

Supplementary Figure 1: LNPs predominantly distributed in the liver.

B6 mice were intravenously injected with LNPs containing 5 μg of FLuc mRNA or 200 μL of PBS (n = 4). The liver, spleen, gastrointestinal tract, kidneys, brain, heart, and lungs were collected 3 h after injection, and bioluminescence was measured. Representative images for each organ are shown. Pictures on the left show photographs of the organs, and those on the right show luminescence. Lv, liver; S, spleen; I, gastrointestinal tract; B, brain; K, kidney; H, heart; Lg, lung.

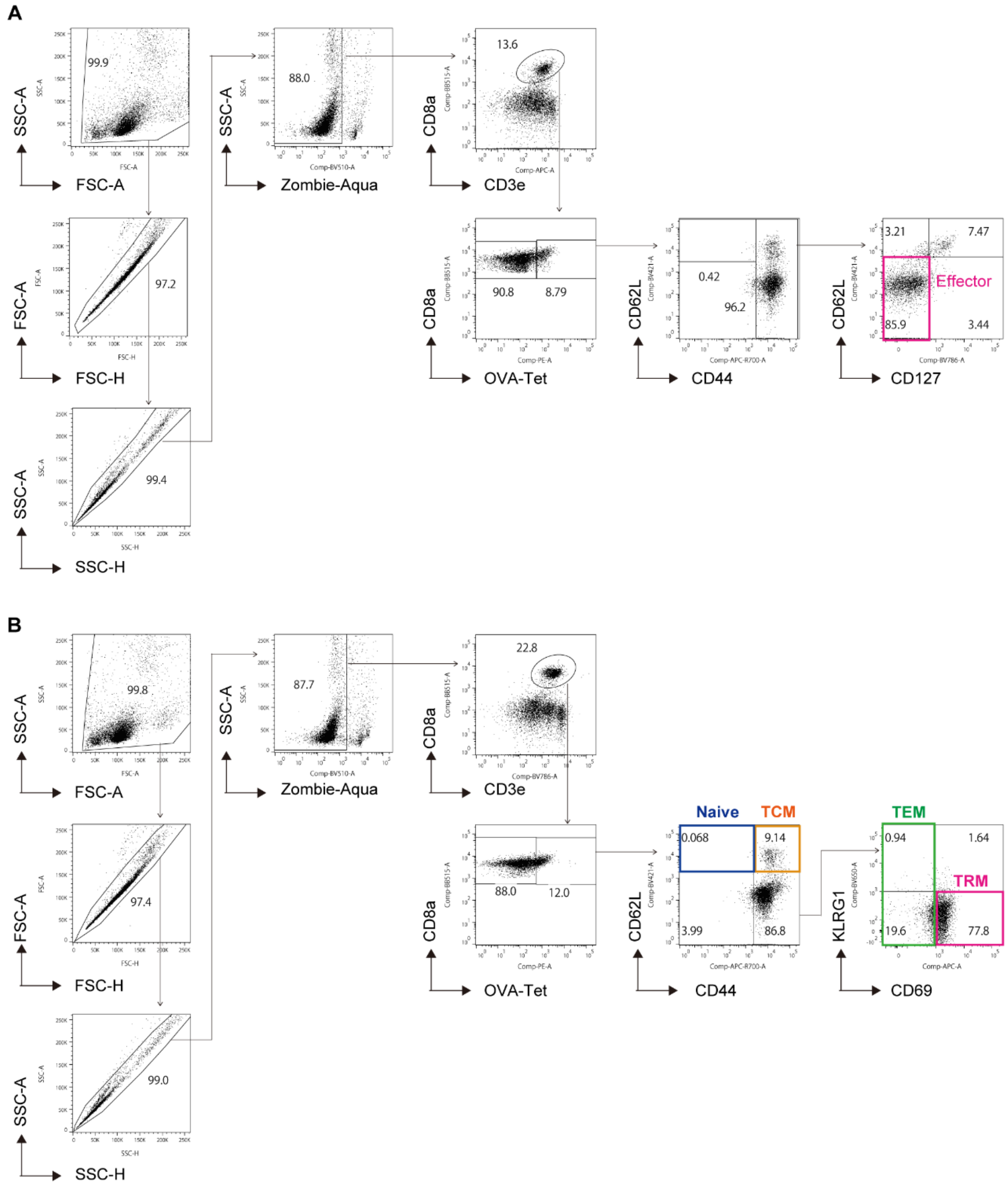

Supplementary Fig. 2

Supplementary Figure 2: Gating strategy of OVA tetramer^{+} CD8^{+} T cells in mice immunized with OVA mRNA-LNPs.

(A) B6 mice were immunized with OVA mRNA-LNPs. Seven days later, liver and spleen cells were analyzed using flow cytometry. The proportion of effector cells characterized by CD44^{hi} CD62L^{lo} CD127^{-} was measured in the OVA tetramer^{+} CD8^{+} T cells.

(B) B6 mice were immunized with OVA mRNA-LNPs. One month later, the liver and spleen cells were analyzed using flow cytometry. The proportions of T_{RM} (CD44^{hi} CD62L^{lo} CD69^{+} KLRG1^{-}), T_{EM} (CD44^{hi} CD62L^{lo} CD69^{-}), T_{CM} (CD44^{hi} CD62L^{hi}), and naïve cells (CD44^{lo} CD62L^{hi}) were measured in the OVA tetramer^{+} CD8^{+} T cells.

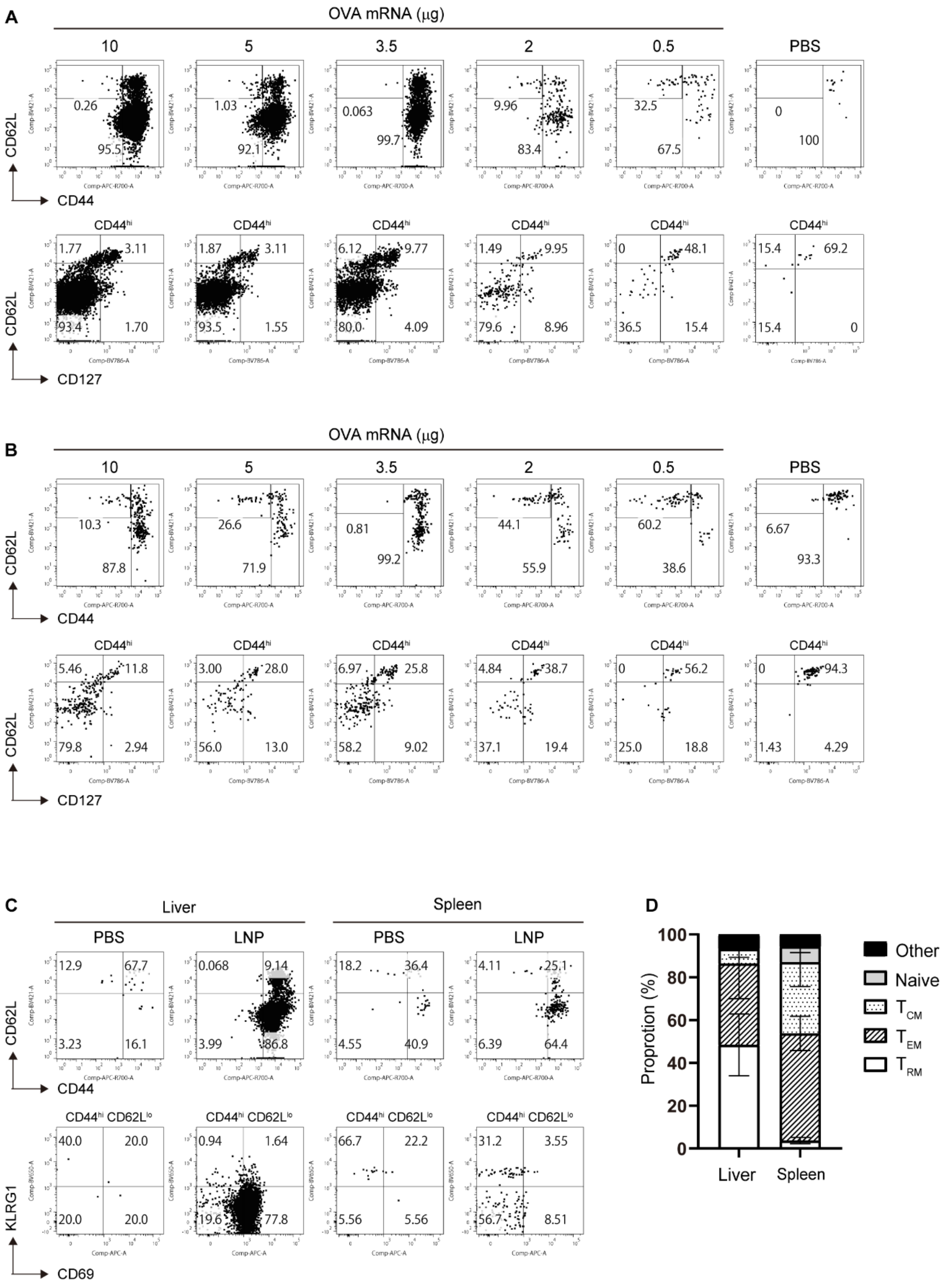

Supplementary Fig. 3

Supplementary Figure 3: OVA mRNA-LNPs induced OVA-specific effector CD8$^+$ T cells and T$_{RM}$ cells predominantly in the liver.

(A, B) B6 mice were intravenously injected with the indicated doses of OVA mRNA-LNPs or 200 µL of PBS. Seven days after injection, the mice were analyzed. The representative dot plots show the expression of CD44 and CD62L in the OVA tetramer$^+$ CD3$^+$CD8$^+$ cells (upper) and that of CD127 and CD62L in the CD44^{hi} OVA tetramer$^+$ CD3$^+$CD8$^+$ cells (lower) within the liver (**A**) and spleen (**B**).

(C) B6 mice were intravenously injected with the indicated doses of OVA mRNA-LNPs or 200 µL of PBS. One month after injection, the mice were analyzed. The representative dot plots show the expression of CD44 and CD62L in the OVA tetramer$^+$ CD3$^+$CD8$^+$ cells (upper) and that of CD69 and KLRG1 in the CD44^{hi} CD62L^{lo} OVA tetramer$^+$ CD3$^+$CD8$^+$ cells (lower) within the liver (left) and spleen (right).

(D) The graph shows the proportion of T$_{RM}$, T$_{EM}$, T$_{CM}$, and naïve cells in the OVA tetramer$^+$ CD8$^+$ T cells summarized in (**C**).

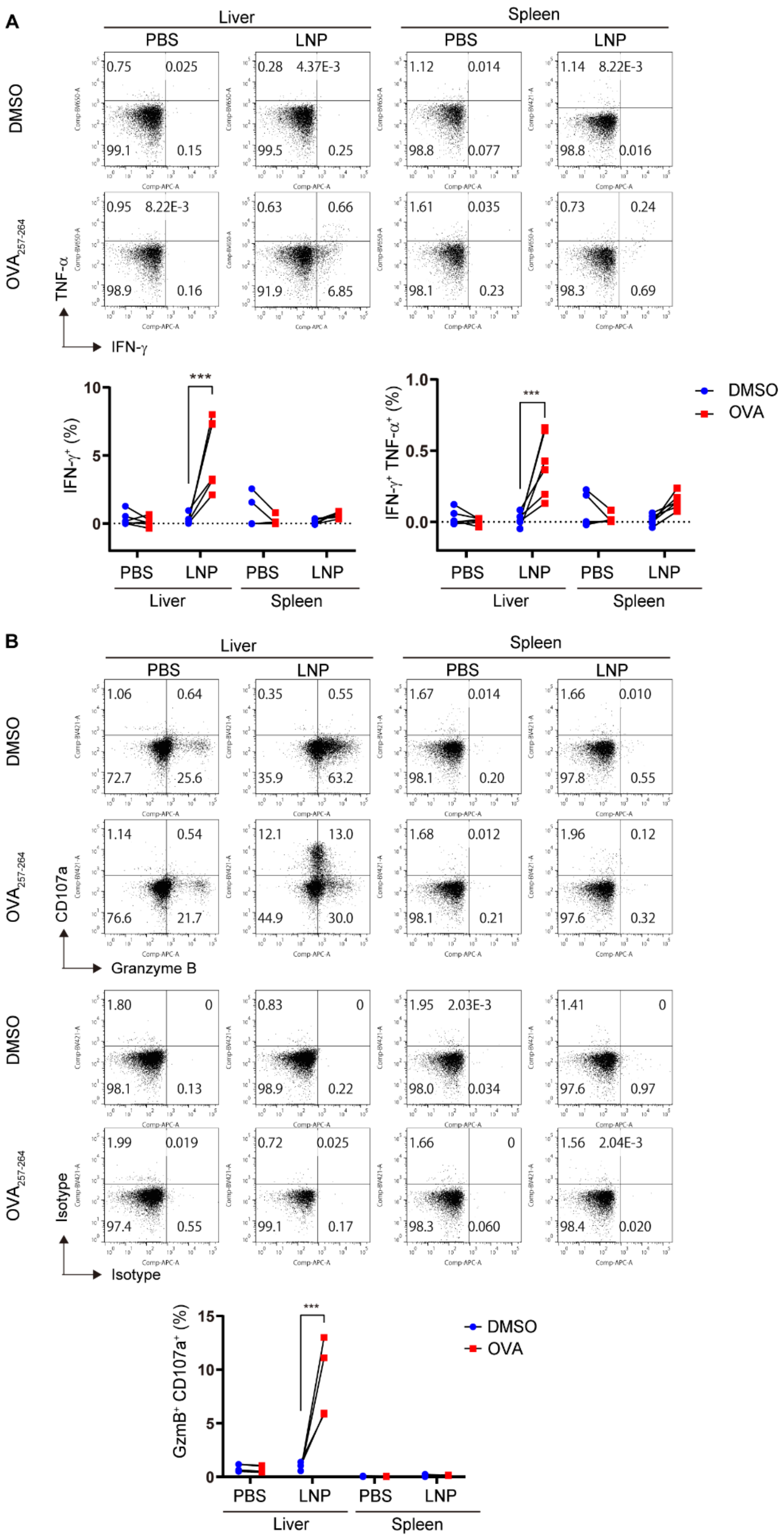

Supplementary Fig. 4

Supplementary Figure 4: Functional analysis of $CD8^+$ T cells from the mice immunized with OVA mRNA-LNPs.

B6 mice were intravenously injected with 5 μg of OVA mRNA-LNPs or 200 μL of PBS. One month after injection, the liver and spleen cells were stimulated with 1 μg/mL of the SIINFEKL peptide for 6 h. (**A**) Representative plot showing the expression of IFN-γ and TNF-α (upper panel) and the proportion of IFN-γ^+ (lower panel) and IFN-γ^+ TNF-α^+ (lower panel) cells within $CD8^+$ T cells in the liver and spleen. (**B**) Representative plot showing the expression of Granzyme B and CD107a (upper) and the proportion of Granzyme B^+ $CD107a^+$ cells within $CD8^+$ T cells in the liver and spleen (below). ***$P < 0.001$ by a two-way ANOVA with Bonferroni's post-hoc test.

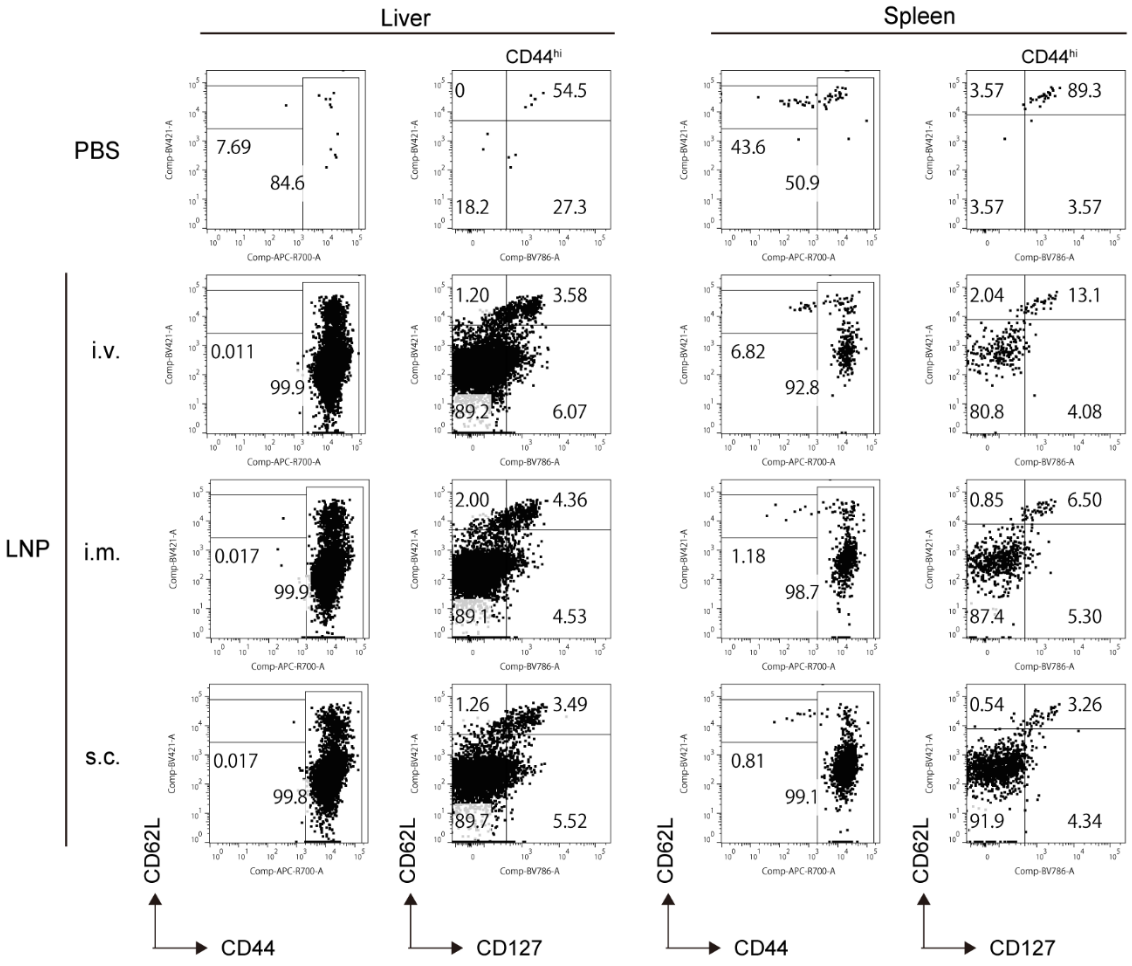

Supplementary Fig. 5

Supplementary Figure 5: The injection routes of the OVA mRNA-LNPs affected the activation of OVA tetramer⁺ CD8 T cells in the spleen but not in the liver.

B6 mice were injected with 5 µg of OVA mRNA-LNPs intravenously (i.v.), intramuscularly (i.m.), or subcutaneously (s.c.; n = 6). As a negative control, another group of mice were intravenously injected with 100 µL of PBS. Seven days after injection, the mice were analyzed. Representative dot plots showing the expression of CD44 and CD62L in the OVA tetramer^{+} CD3^{+}CD8^{+} cells (right) and that of CD127 and CD62L in the CD44^{hi} OVA tetramer^{+} CD3^{+}CD8^{+} cells (left) within the liver.

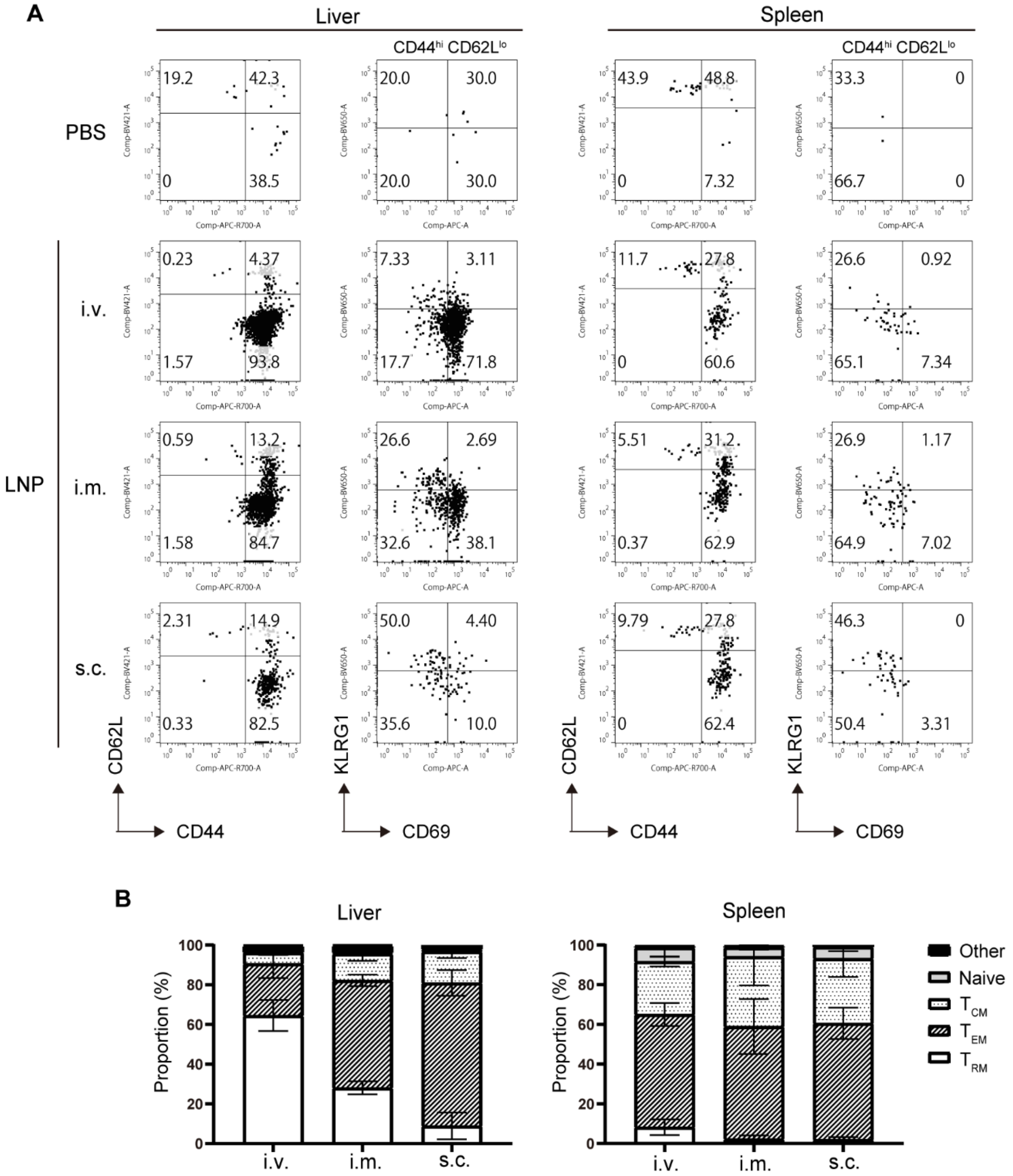

Supplementary Fig. 6

Supplementary Figure 6: The injection routes of the OVA mRNA-LNPs affected the generation of memory cells in the liver.

(A, B) B6 mice were injected with 5 µg of OVA mRNA-LNPs intravenously (i.v.), intramuscularly (i.m.), or subcutaneously (s.c.; n = 6). As a negative control, another group of mice were intravenously injected with 100 µL of PBS. One month after injection, the mice were analyzed. (**A**) Representative dot plots showing the expression of CD44 and CD62L in the OVA tetramer^{+} CD3^{+}CD8^{+} cells (right) and that of CD69 and KLRG1 in the CD44^{hi} CD62L^{lo} OVA tetramer^{+} CD3^{+}CD8^{+} cells (lower) within the liver and spleen. (**B**) The graph shows the proportion of T_{RM}, T_{EM}, T_{CM}, and naïve cells in the OVA tetramer^{+} CD8^{+} T cells summarized in (**A**).

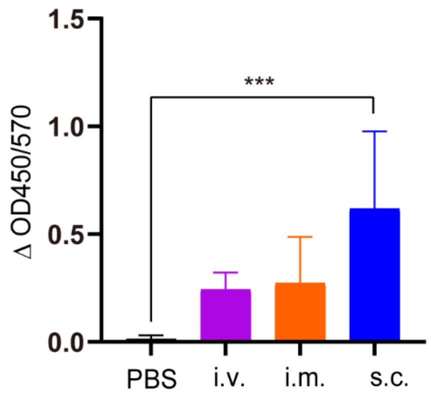

Supplementary Fig. 7

Supplementary Figure 7: Anti-OVA-specific IgG titers in mice immunized with OVA mRNA-LNPs.

B6 mice were injected with 5 µg of OVA mRNA-LNPs intravenously (i.v.), intramuscularly (i.m.), or subcutaneously (s.c.; n = 6). As a negative control, another group of mice were intravenously injected with 100 µL of PBS. One month after injection, sera were collected, and the anti-OVA-specific IgG titers were measured. Data represent the mean ± SD; ***$P < 0.001$ by a one-way ANOVA with Bonferroni's post-hoc test.

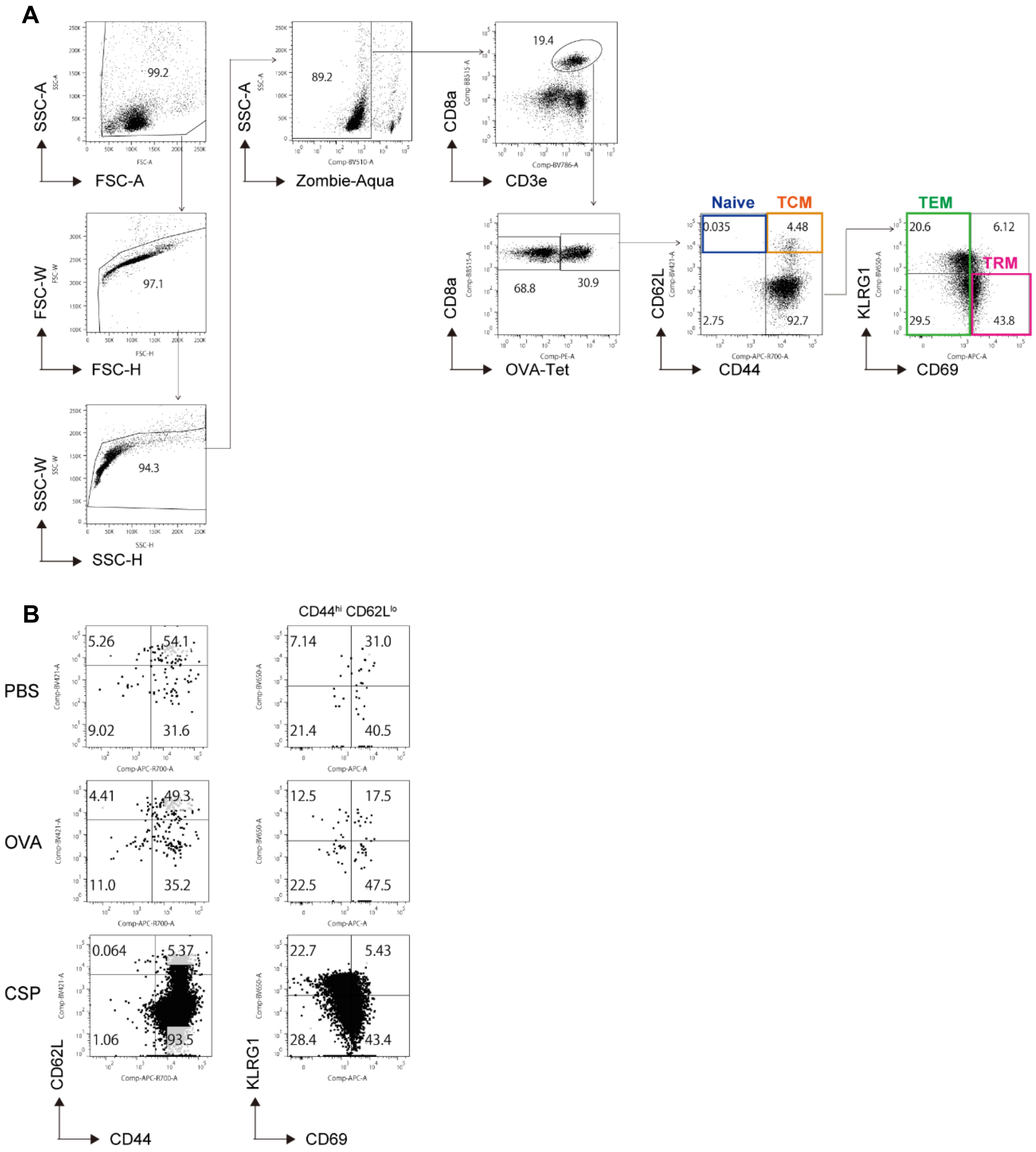

Supplementary Fig. 8

Supplementary Figure 8: Two intramuscular injections of CSP mRNA-LNPs induced CSP tetramer^{+} T_{RM} cells in the liver.

(A) The gating strategy is shown. BALB/c mice were immunized with CSP mRNA-LNPs. One month later, the liver and spleen cells were analyzed by flow cytometry. The proportions of T_{RM} (CD44^{hi} CD62L^{lo} CD69^{+} KLRG1^{-}), T_{EM} (CD44^{hi} CD62L^{lo} CD69^{-}), T_{CM} (CD44^{hi} CD62L^{hi}), and naïve cells (CD44^{lo} CD62L^{hi}) were measured in the CSP tetramer^{+} CD8^{+} T cells.

(B) BALB/c mice were intramuscularly injected with 3.35 μg of CSP mRNA-LNPs twice at 3-week intervals (n = 3). As a negative control, another group of mice were injected with 100 μL of PBS or 5 μg of OVA mRNA-LNPs in the same manner. One month after the final injection, the mice were analyzed. Representative dot plots showing the expression of CD44 and CD62L in the CSP tetramer^{+} CD3^{+}CD8^{+} cells (right) and that of CD69 and KLRG1 in the CD44^{hi} CD62L^{lo} CSP tetramer^{+} CD3^{+}CD8^{+} cells (lower) within the liver and spleen.

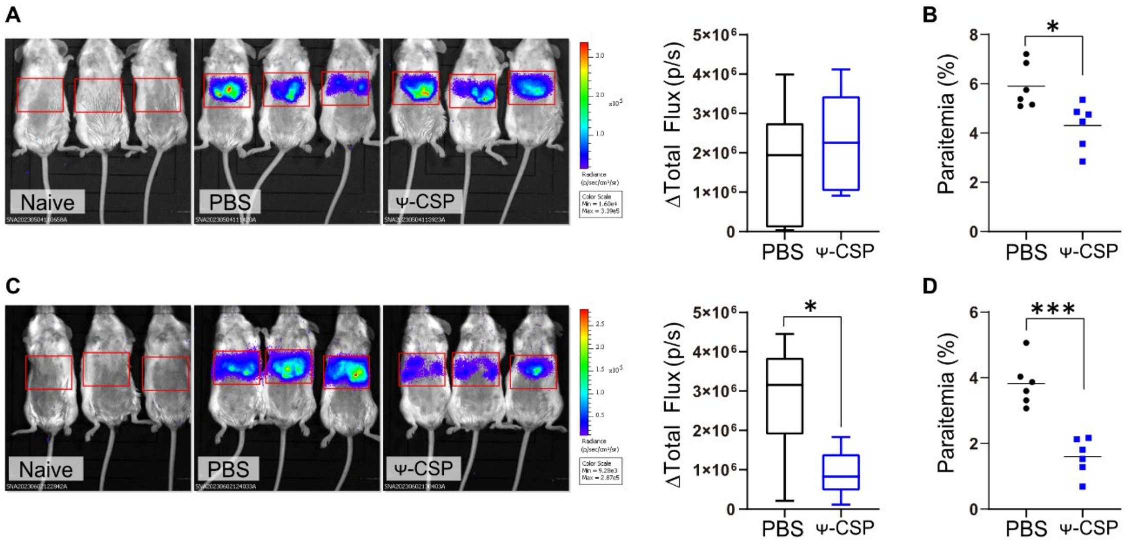

Supplementary Fig. 9

Supplementary Figure 9: Longevity of protective immunity induced by CSP mRNA-LNPs.

BALB/c mice were intramuscularly injected with 6.7 µg of N1-methyl-pseudouridine CSP (Ψ-CSP) mRNA-LNPs twice at 3-week intervals (n = 6). As a negative control, another group of mice were injected with 100 µL of PBS in the same manner. Nine weeks (**A, B**) and 13 weeks (**C, D**) after the final immunization, the mice were infected with 3000 of luciferase and GFP expressing *P. berghei* sporozoites. (**A, C**) Bioluminescence was measured 44 h post infection. Representative images of ventrodorsal shootings are shown on the left. A summary graph of the total flux [photons/s] in the liver is shown on the right. (**B, D**) Parasitaemia 7 days (**B**) and 8 days (**D**) after infection is shown.

These results were obtained across two experiments. The data represent the mean ± SD (**A, C**) or the mean (**B, D**); *$P < 0.05$, ***$P < 0.001$, as indicated by Welch's t-test.
